# Real-time dialogue between experimenters and dreamers during REM sleep

**DOI:** 10.1016/j.cub.2021.01.026

**Published:** 2021-02-18

**Authors:** Karen R. Konkoly, Kristoffer Appel, Emma Chabani, Anastasia Mangiaruga, Jarrod Gott, Remington Mallett, Bruce Caughran, Sarah Witkowski, Nathan W. Whitmore, Christopher Y. Mazurek, Jonathan B. Berent, Frederik D. Weber, Başak Türker, Smaranda Leu-Semenescu, Jean-Baptiste Maranci, Gordon Pipa, Isabelle Arnulf, Delphine Oudiette, Martin Dresler, Ken A. Paller

**Affiliations:** 1 Department of Psychology and Cognitive Neuroscience Program, Northwestern University, Evanston, IL, USA; 2 Institute of Cognitive Science, Osnabrück University, Osnabrück, Germany; 3 Institute of Sleep and Dream Technologies, Hamburg, Germany; 4 Institut du Cerveau - Paris Brain Institute - ICM, Sorbonne Université, Inserm, CNRS, Paris, France; 5 Donders Institute for Brain, Cognition and Behavior, Radboud University Medical Center, Nijmegen, the Netherlands; 6 Department of Medical and Surgical Sciences, University of Bologna, Bologna, Italy; 7 Department of Psychology, University of Texas at Austin, Austin, TX, USA; 8 NextSense, Inc., Mountain View, CA, USA; 9 AP-HP, Pitié -Salpêtrière Hospital, Sleep Disorders Department, Paris, France; 10 Twitter: @kap101; 11 These authors contributed equally; 12 These authors contributed equally; 13 Lead contact

## Abstract

Dreams take us to a different reality, a hallucinatory world that feels as real as any waking experience. These often-bizarre episodes are emblematic of human sleep but have yet to be adequately explained. Retrospective dream reports are subject to distortion and forgetting, presenting a fundamental challenge for neuroscientific studies of dreaming. Here we show that individuals who are asleep and in the midst of a lucid dream (aware of the fact that they are currently dreaming) can perceive questions from an experimenter and provide answers using electrophysiological signals. We implemented our procedures for two-way communication during polysomnographically verified rapid-eye-movement (REM) sleep in 36 individuals. Some had minimal prior experience with lucid dreaming, others were frequent lucid dreamers, and one was a patient with narcolepsy who had frequent lucid dreams. During REM sleep, these individuals exhibited various capabilities, including performing veridical perceptual analysis of novel information, maintaining information in working memory, computing simple answers, and expressing volitional replies. Their responses included distinctive eye movements and selective facial muscle contractions, constituting correctly answered questions on 29 occasions across 6 of the individuals tested. These repeated observations of interactive dreaming, documented by four independent laboratory groups, demonstrate that phenomenological and cognitive characteristics of dreaming can be interrogated in real time. This relatively unexplored communication channel can enable a variety of practical applications and a new strategy for the empirical exploration of dreams.

## In Brief

Scientific investigations of dreaming have been hampered by the delay between a dream and when people report on their dream, and by a change in state from sleep to wake. To overcome this problem, Konkoly et al. show that individuals in REM sleep can perceive and answer an experimenter’s questions, allowing for real-time communication about a dream.

## Graphical Abstract

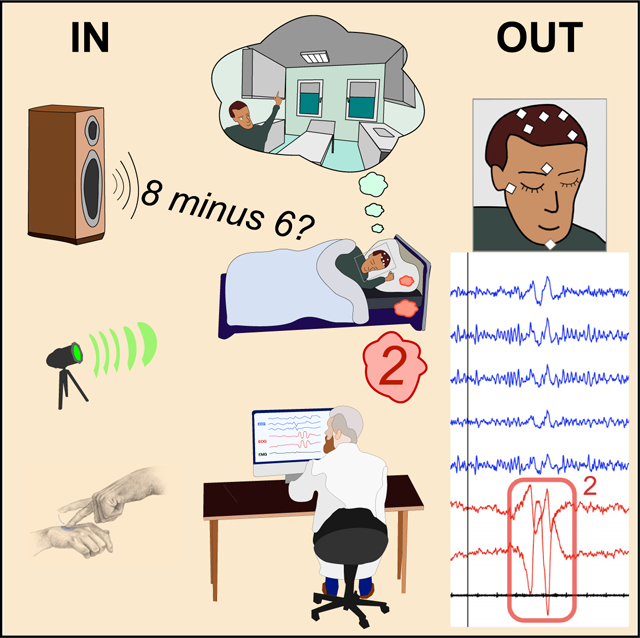

## INTRODUCTION

Why do we have dreams? How are dream scenarios created? Does dreaming confer any benefit for brain function? These and other questions have remained open,^1^ in part, because of the limited options available for peering into dream experiences. Dream reports given after waking tend to be distorted or fragmentary due to our generally poor ability to form new memories in the sleep state and the limited capacity we have to accurately keep recent information in mind after the dream has ended. There is considerable ambiguity about the nature and timing of experiences that may have transpired during a dream, as revealed through retrospective reporting. The ability to communicate with dreamers in real time, such that they could describe their experiences while in the midst of a dream, would greatly expand the possibilities for scientifically exploring dream experiences.

Putative neural signals of dream content have been acquired by several groups based on dream reports produced shortly after waking.^2–5^ This neural decoding has been accomplished using a combination of electrical and hemodynamic brain imaging. Horikawa and colleagues^2^ studied the dreamlike experiences of stage 1 hypnagogic imagery, and Dresler and colleagues^3^ studied dreaming during REM (rapid eye movement) sleep. Similarly, Siclari and colleagues^4,5^ used high-density scalp EEG (electro-encephalography) to show that dream reports were associated with a reduction in posterior slow-wave activity during both REM and non-REM sleep stages. Furthermore, the scalp topography of 25–50 Hz EEG activity was found to correspond with aspects of dream content such as spatial experiences and movement. Further studies along these lines could be more informative if conducted in conjunction with real-time data on the subjective experience of dreaming.

Instead of waiting for dreamers to tell us about a dream after it has ended, when they have transitioned to the waking state, we sought to obtain evidence showing that it is possible to interview them about their dreams at the time they are experiencing them. Our experimental goal is akin to finding a way to talk with an astronaut who is on another world, but in this case the world is entirely fabricated on the basis of memories stored in the brain. Demonstrating the viability of this “interactive dreaming”—when experimenter and dreamer communicate with each other in real time—would be a large step forward to promote future progress in dream research.

In typical dreams, people judge their experience with a high degree of acceptance and a lack of critical evaluation; they fail to realize that their experience is merely a dream. On the other hand, a “lucid dream” differs in that the dreamer gains the elusive insight of being in a dream.^6,7^ Lucid dreams occur pre-dominantly during REM sleep and can be accompanied by eye-movement signals used to indicate that dreamers recognize that they are dreaming^8,9^ or to transmit other information such as time-stamping dream events.^10,11^ However, lucid dreaming is a notoriously rare phenomenon and lucid dreams can seldom be summoned at will, which has made it difficult for researchers to capture them in the lab in a reliable manner.

**Figure 1.**
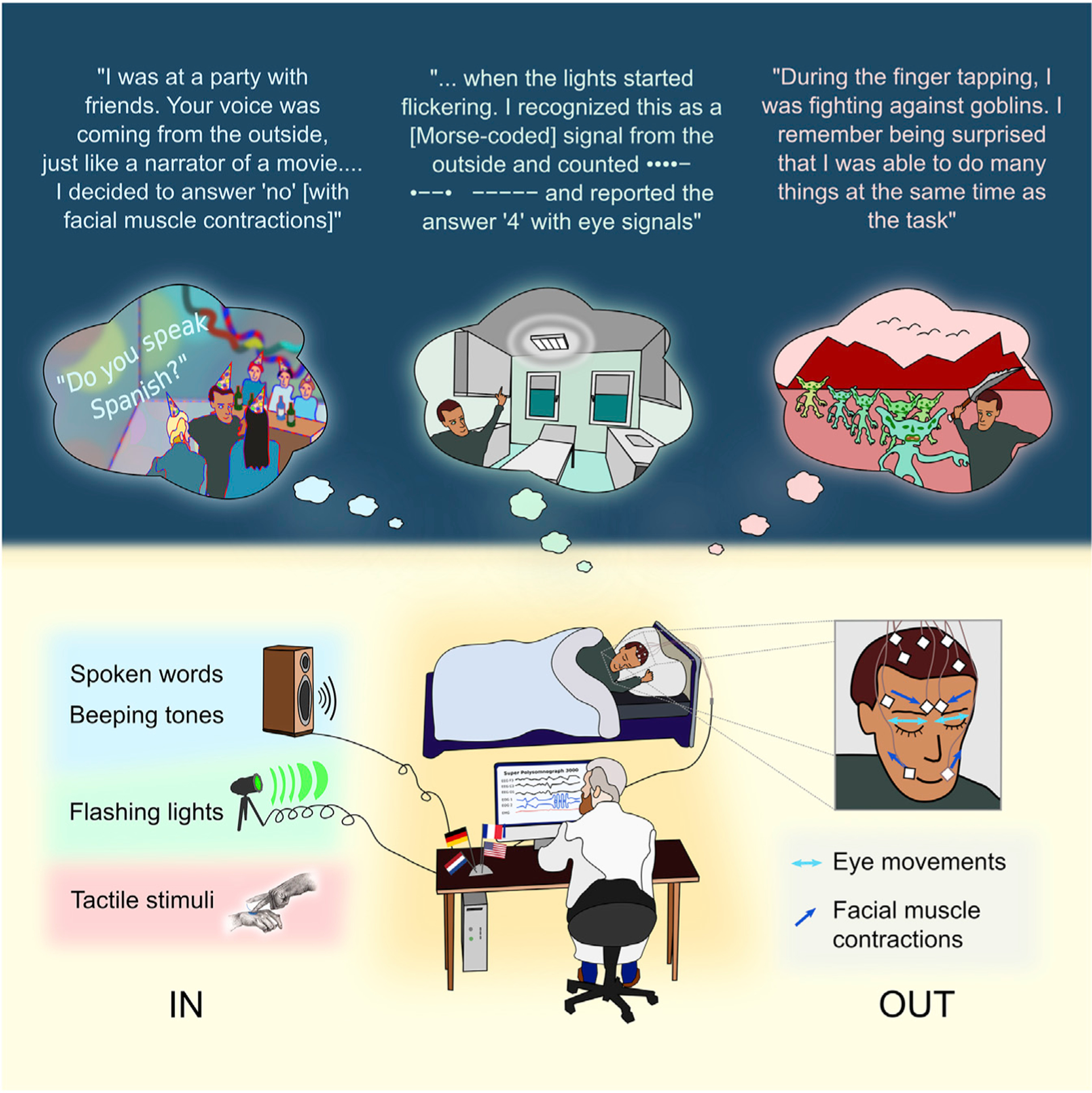
Overview of the experimental setting IN (lower left) refers to methods whereby information was transmitted from experimenter to dreamer. OUT (lower right) refers to methods whereby information was transmitted from dreamer to experimenter. Examples of three dreams (color-coded for each input method) are illustrated below relevant excerpts from corresponding dream reports obtained following awakening.

Here, we report multiple demonstrations of successful two-way communication during lucid dreams achieved by four independent scientific teams in France, Germany, the Netherlands, and the USA. We substantiate the validity of this interactive-dreaming phenomenon by bringing together results obtained using a diverse set of strategies. Several methods for communicating into and out of dreams were used, as shown in Figure 1. Lucid dreamers were able to follow instructions to compute mathematical operations, answer yes-or-no questions, or discriminate stimuli in the visual, tactile, and auditory modalities. They were able to respond using volitional control of gaze direction or of different facial muscles. There were three different participant categories: (1) experienced lucid dreamers, (2) healthy people with minimal prior experience who we trained to lucid dream, and (3) a patient with narcolepsy, a neurological disorder characterized by excessive daytime sleepiness, short-latency REM sleep periods, and frequent lucid dreaming. Evidence of two-way communication was found with all three participant categories, and also with both nocturnal sleep and daytime naps.

Various strategies for influencing dreams and/or memory storage during sleep have been examined in the past, as recently reviewed by Oudiette and Paller.^12^ In such studies, participants process external cues while remaining asleep but do not communicate back while asleep. Interestingly, a recent study by Strauss and Dehaene^13^ focused on electro- and magnetoencephalographic responses to spoken arithmetic equations (addition, multiplication, or subtraction operations). Differential N400 and P600 responses to correct versus incorrect equations were elicited during attentive wakefulness, but mostly absent during N2 and REM sleep, which led the authors to conclude that “the explicit computation of the arithmetic result is lost during sleep” (p. 10). If given a mathematical *question* instead, could sleeping people answer? Contemporary research on sensory stimulation during sleep, with notable exceptions,^14–16^ has largely proceeded without the goal of eliciting volitional responses during sleep. For example, electrical responses from the brain during sleep have been investigated in many studies using the individual’s own spoken name and other stimuli, but without any interactions that could be construed as two-way communication.^17–23^ Whereas the idea of communicating interactively with sleeping individuals may seem outlandish, the legitimacy of this phenomenon is strongly supported by the following examples of successful two-way communication.

## RESULTS

The four research groups each established bidirectional communication using somewhat different procedures, as described below. In each case, REM sleep was verified with standard polysomnographic methods, and sensory stimulation was used to convey questions to the dreaming participant. Many participants first produced a pre-arranged ocular response (a series of left-right eye signals) to indicate that they were experiencing a lucid dream. Importantly, our procedures involved training prior to sleep with the same type of sensory stimulation used during sleep. We also included training with response methods. Note that automated responses were unlikely given the effort required to translate answers to signals. Participants generally practiced receiving questions from the experimenter and producing answers in the form of physiological signals based on facial or eye movements. Yet participants did not know which specific questions would be presented to them during sleep, such that the communication subsequently undertaken during sleep was always novel.

**Figure 2.**
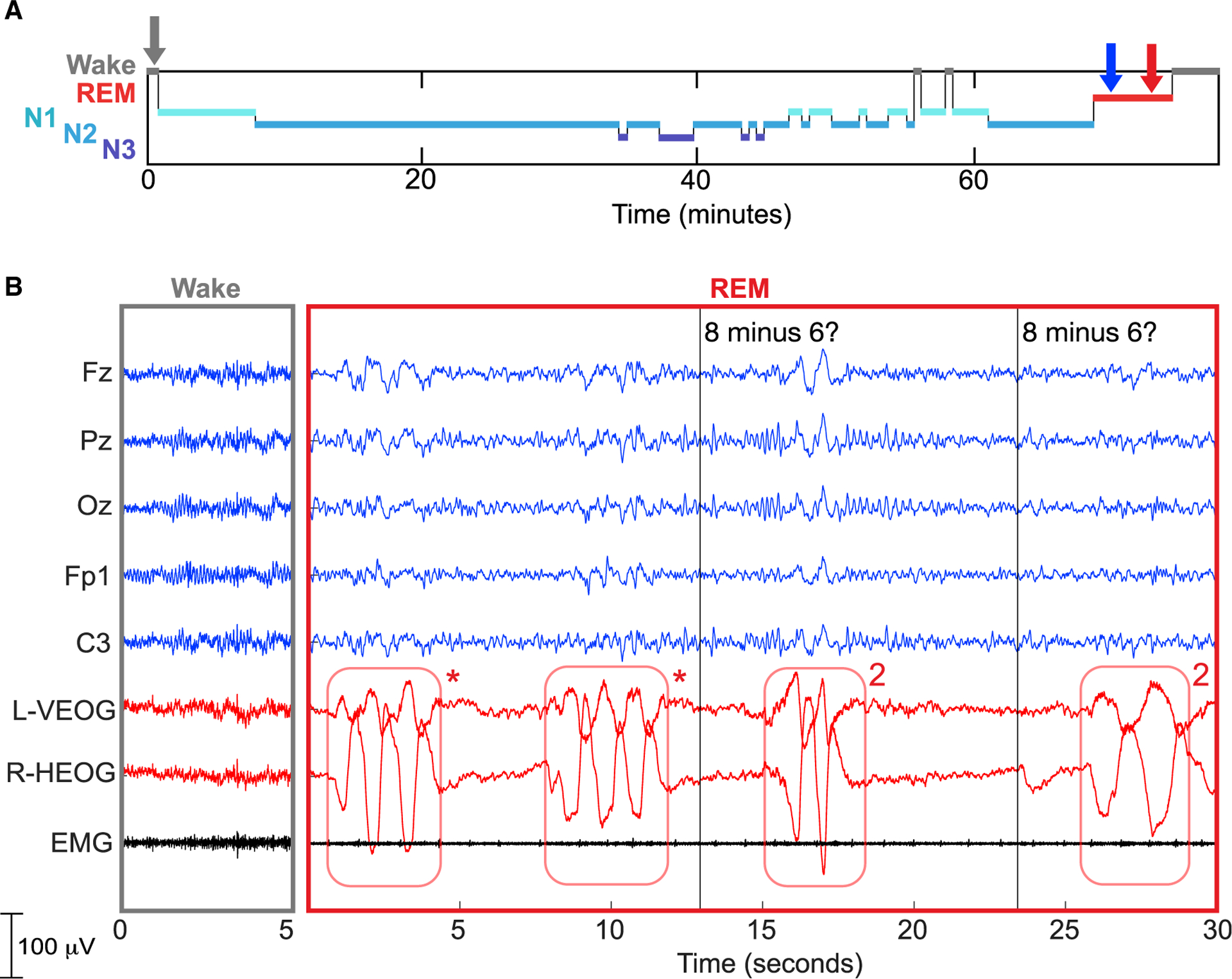
Interactive dreaming (USA group) (A) Hypnogram showing that REM sleep began 68 min after sleep onset. The auditory cue to induce lucidity was presented two times (blue arrow), followed by a microarousal and then a longer REM period with lucidity signals (LRLRLR) given six times starting at 69 min. (B) The left panel shows a 5 s period of wake, corresponding to the gray arrow on the hypnogram. The right panel shows a 30 s REM segment, in which the last two lucidity signals (indicated by red asterisks) were followed by two instances of the spoken stimulus “8 minus 6” (vertical lines, and red arrow in A). Both times, the correct answer was produced with eye signals (2). Upon awakening, the participant reported dreaming about his favorite video game: “I was in a parking lot at night…then suddenly it was daytime and I was in the video game…. I thought, okay this is probably a dream. And then something weird…. I lost control of all my muscles. There was a roaring sound of blood rushing to my ears.” The experimenter asked him whether he remembered hearing any math problems, how many he answered, and what he answered. The subject reported, “I think I heard three [problems]…. I answered ‘2’ for all of them, but I don’t remember what the first one was. I just remember the last one was ‘8 minus 6.”’ (For further details on sleep monitoring and terminology, see Nir and Tononi,^1^ Appel et al.,^6^ and Baird et al.^7^)

Data in Figure 2 were obtained from a 19-year-old American participant who reported experiencing only two lucid dreams previously. He received sound cues during a 90-min daytime nap, near the beginning of a period of REM sleep. He indicated that he was in a lucid dream with a series of three left-right eye movements (termed LRLRLR). Then we presented a spoken math problem: 8 minus 6. Within 3 s, he responded with two left-right eye movements (LRLR) to signal the correct answer 2. The math problem was then repeated, and he again produced the correct answer. Note that participants were instructed to make rapid eye movements with a maximal horizontal scan, yielding EOG signals (as in this case) that strikingly stand out from typical eye movements during REM sleep.

**Figure 3.**
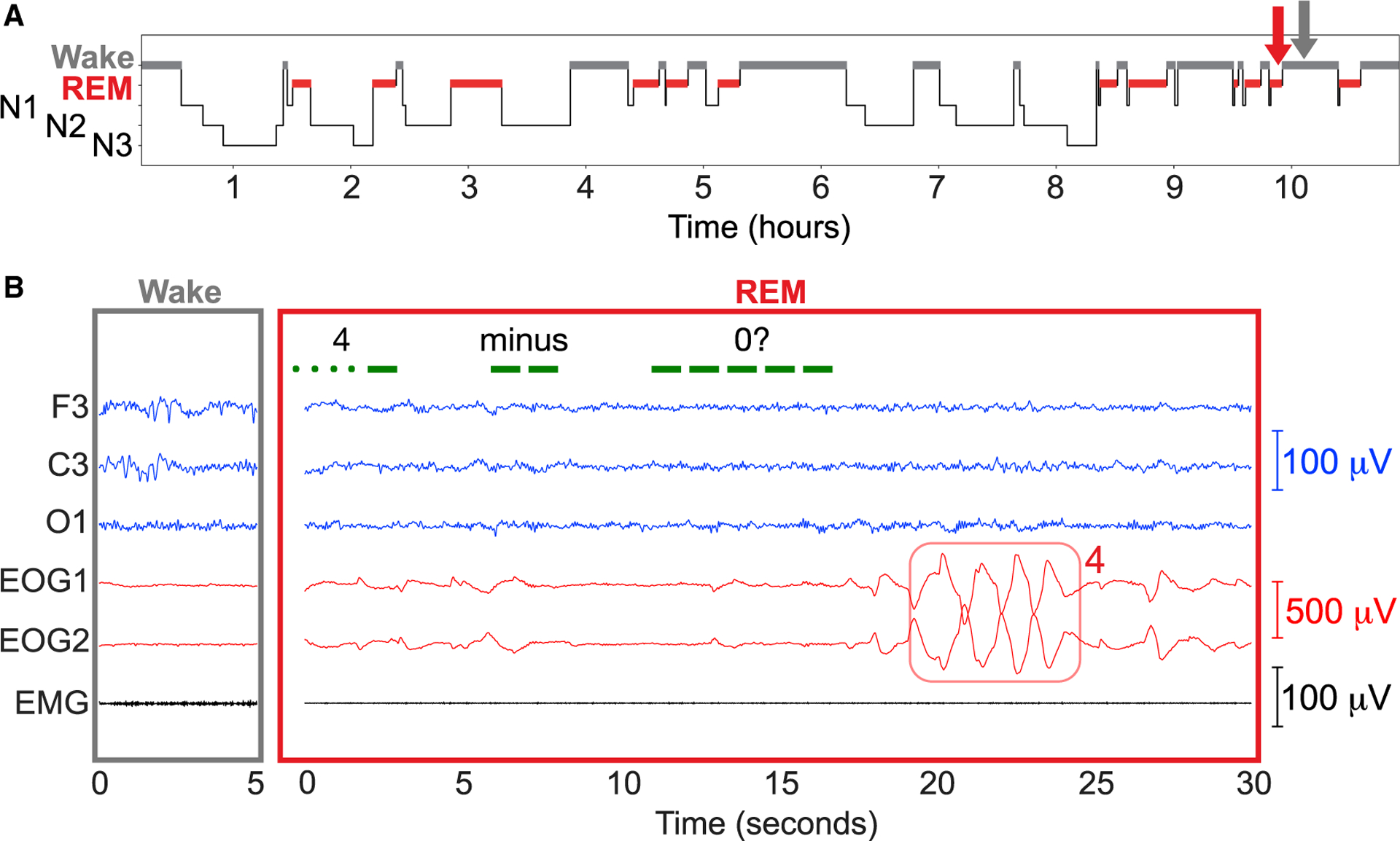
Interactive dreaming (German group) The participant was stimulated during REM sleep with red and green LED light flashes to convey Morse-coded math problems. (A) Hypnogram of the night. (B) An awake period (left) and a period of interactive dreaming during REM sleep (right), corresponding to the times indicated by the gray and red arrows in (A), respectively. The question “4 minus 0” was presented, as shown in green. The resulting answer “4” produced by the dreamer was apparent in the EOG signals. Upon awakening, the participant recalled the problem almost correctly. Dream report: “A medical practice, maybe for physiotherapy. I was alone in the room and there was a large doctor’s couch in the middle of the room, shelves, sideboards. The couch was strange. The room seemed solid and steady, when the lights started flickering. I recognized this as the flashing signal [Morse code] from the outside (4 plus 0, 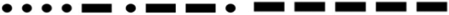) and reported the answer ‘4’ with eye signals. I looked for a tool that could flash, and I found a round bowl full of water. The water flashed (like a fish tank light that one turns on and off). I again saw a signal, but was not able to identify it. The bowl broke because I accidentally let it fall while trying to decode the flashes. I left the room, trying to find something else that could flash, and went outside and looked up to the clouds. There was yellow sunlight and light gray clouds. I saw variations in the brightness, clouds drifting past quickly, but again, unfortunately, I could not decipher a flashing signal. It was too fast to decode, but I knew that these were math problems.”

The following three additional examples also document dreamers and experimenters in conversation. Figure 3 shows results from a 35-year-old German participant who was an experienced lucid dreamer. After a lucidity signal was observed during nighttime REM sleep, we presented visual stimuli consisting of alternating colors and corresponding to a Morse-coded math problem “4 minus 0.” The participant produced the correct answer “4” using left-right eye movements (LRLRLRLR). In his description of the dream, he maintained that he heard the message “4 plus 0” and answered accordingly.

**Figure 4.**
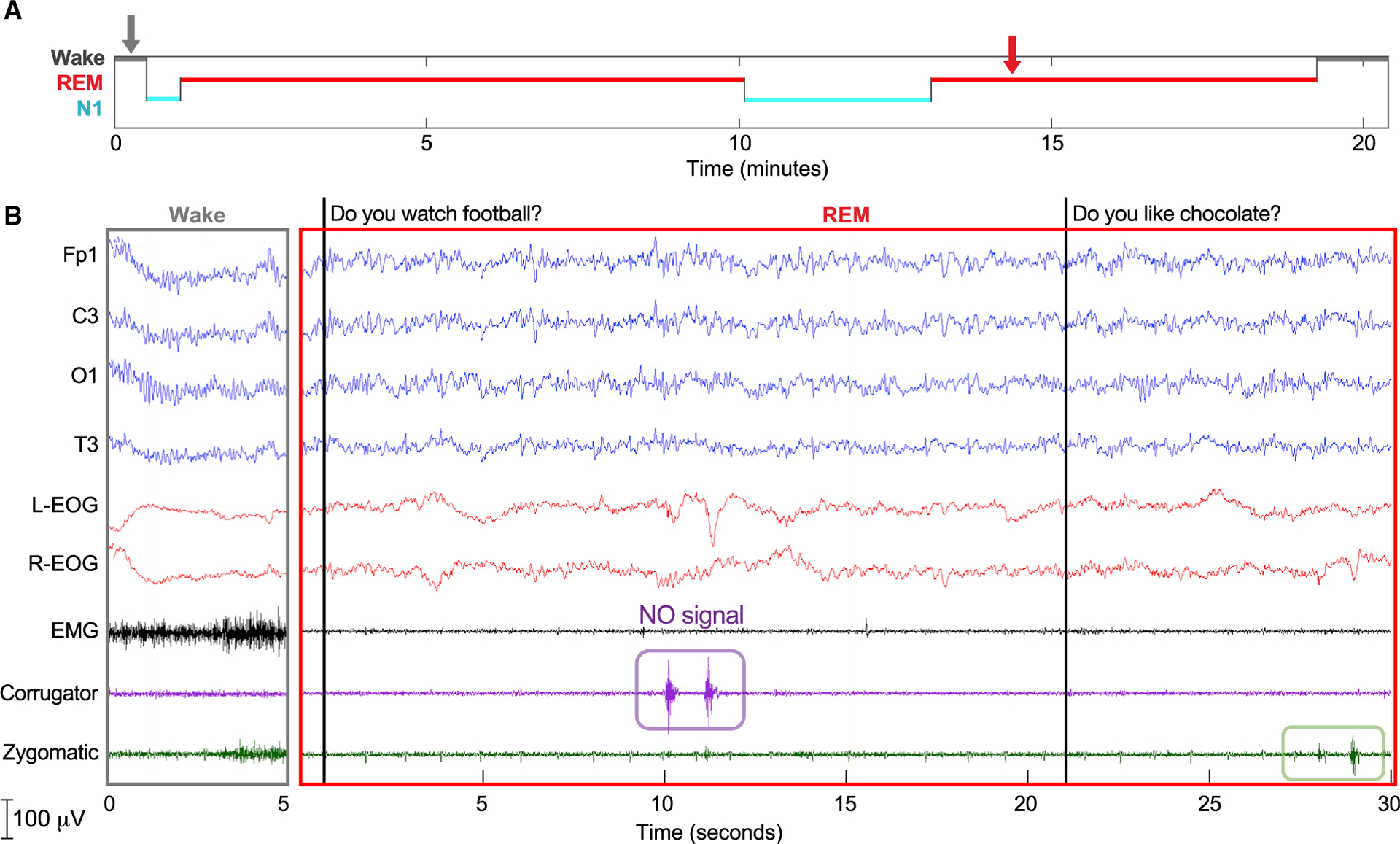
Interactive dreaming (French group) (A) Hypnogram showing a daytime nap in a participant with narcolepsy. The red arrow indicates the beginning of a yes-no question period. Before sleep, the participant was instructed to contract zygomatic muscles twice to signal “YES” and corrugator muscles twice to signal “NO.” (B) Polysomnographic results documenting periods of wake (left) and REM sleep from the beginning of a yes-no question period (right). The first question was answered correctly (NO signal). The next question was answered, but the answer was judged as ambiguous. Three further questions were asked. In total, four of these five questions were answered; negligible facial EMG activity was observed after one question. Two answers were rated as correct and two as ambiguous. There was no facial EMG activity outside of the stimulation periods. The dream report upon waking was as follows: “In my dream, I was at a party and I heard you asking questions. I heard your voice as if you were a God. Your voice was coming from the outside, just like a narrator of a movie. I heard you asking whether I like chocolate, whether I was studying biology, and whether I speak Spanish. I wasn’t sure how to answer the last one, because I am not fluent in Spanish, but I have some notions. In the end, I decided to answer ‘NO’ and went back to the party.”

Figure 4 shows results from a 20-year-old French participant with narcolepsy and remarkable lucid-dreaming abilities. Because of his narcolepsy, he reached REM sleep quickly, about 1 min after the beginning of a 20-min daytime nap, and he signaled lucidity 5 min later. We verbally asked him yes/no questions and he answered correctly using facial muscle contractions (zygomatic muscle for yes, corrugator muscle for no). In a separate analysis of facial contractions during lucid dreaming, we never observed a response in the absence of stimulation.

**Figure 5.**
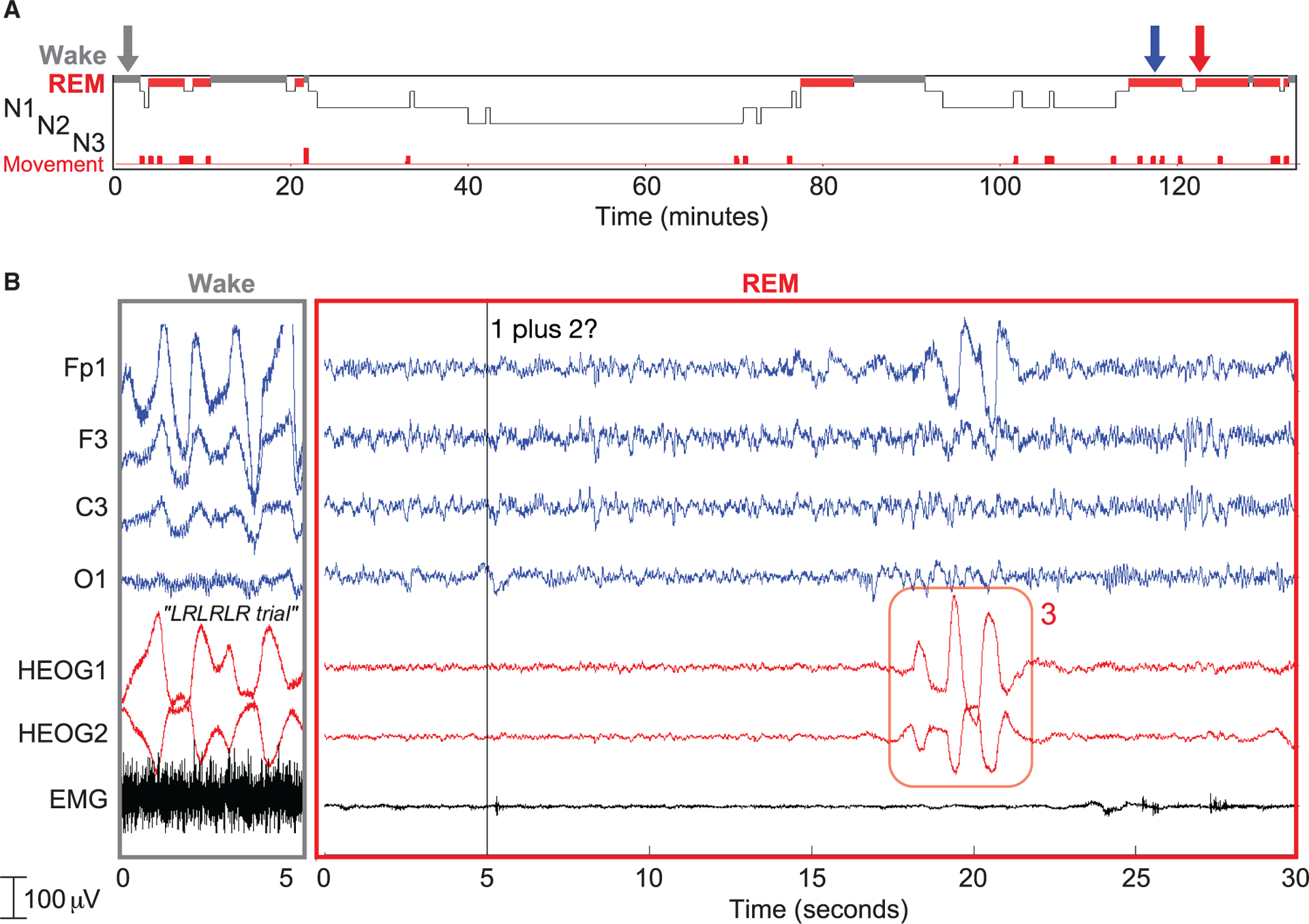
Interactive dreaming (Dutch group) (A) Hypnogram of the nap. The blue arrow indicates the third instance when auditory and visual cues for lucid-dream induction were administered. We administered 24 math problems but refrained from immediately awakening this participant for a dream report due to highly fragmented REM sleep, with many stage N1 intrusions and movement arousals (indicated by red at bottom). (B) A period of wake with an LRLRLR signal (left, gray arrow in A) and a period of REM (right, red arrow in A). The math problem in this example (1 plus 2) was the seventh problem delivered and was followed by a correct eye-movement response (3). Dream report: “…in my dream I thought ‘I have to remember things’ and I heard the sounds and heard you talking while I was dreaming. I sat down in the car, and then I got a part of the assignment…. I was also really proud that I succeeded with a sum calculation, and that I heard them, and that I was aware that I was dreaming.” The participant stated that the source of the math problems “felt like a sort of radio in the car.”

Figure 5 shows results from a 26-year-old Dutch participant, cued with auditory and visual cues during a 134-min morning nap. Although the participant did not give a lucid signal before the two-way communication attempt (thus excluding this trial from the final count of attempts), she nevertheless answered two math problems correctly and three incorrectly, and she reported a lucid dream upon awakening. In this example, we presented the spoken math problem “1 plus 2” and about 14 s later she produced eye signals to indicate the answer “3.”

Our general approach was to awaken the participant from sleep after achieving successful two-way communication, in order to obtain a dream report. The essential evidence of communication between experimenters and dreamers is documented in physiological recordings such as those shown in Figures 2, 3, 4, and 5. These recordings document (1) REM sleep during the period of communication, as assessed by the experimenter and by a group of independent experts; (2) a marker for the time of the experimenter’s query; and (3) subsequent signals of a participant’s correct answer. A correspondence between this documented communication and a dream report can be taken as additional substantiation of volitional communication on the part of the participant.

Indeed, participants typically reported that they had received experimenters’ questions in their dreams. After some dreams, however, the events of communication were not recalled or were recalled in a distorted manner. Interestingly, participants reported that some signals were received as if coming from outside the dream or superimposed over the dream, whereas other signals were transmitted through components of the dream. For example, some words were heard as if played through a radio or delivered through means available during the dream. Moreover, details of communication that were recalled in dream reports sometimes diverged from the recordings made during the dream. For example, participants sometimes reported a math problem differing from the one presented or an answer differing from the response that was registered. This divergence underscores the difficulty of investigating dreaming by relying on dream reports alone. The transition to the waking state and the time that has elapsed may both contribute to producing a dream report that is not always a veridical reflection of what happened during the dream.

In total, 36 individuals participated in our two-way communication protocols. Table 1 summarizes differing procedures and results across the four teams. In total, we attempted two-way communication during REM sleep in 57 sessions (each nap was counted as one session for the American, French, and Dutch teams, but because there were multiple awakenings overnight for the German team, each bout of sleep during which stimulation took place was considered one session for the purpose of quantifying communication attempts here). In 26% of these sessions, participants successfully signaled to indicate that they were in a lucid dream. In 47% of these signal-verified lucid-dreaming episodes, we obtained at least one correct response to an experimental query. We attempted to communicate with the dreamer on a total of 158 occasions during signal-verified lucid dreams. Table 2 provides a breakdown of the outcomes. Across all teams, we observed a correct response on 18.4% of these trials; the independent experts unanimously scored the polysomnographic evidence as indicating REM sleep for 26 of these 29 trials. On a further 17.7% of the trials, expert raters did not agree on deciphering the response (and on 9 of those trials two raters thought there was no response). An incorrect response was produced on 3.2% of the trials. The most common outcome was a lack of a response (60.1% of the trials).

On two occasions we observed a correct response when attempting two-way communication during REM sleep without a previous lucidity signal but with a subsequent dream report describing the experience of lucidity (one example is in Figure 5). On 379 trials we attempted two-way communication when there was neither a lucidity signal during sleep nor a dream report of lucidity subsequently (32 trials USA; 347 trials Germany). During these non-lucid REM sleep trials, we observed 1 correct response, 1 incorrect response, 11 ambiguous responses, and 366 trials with no response. The fact that response signals were exceedingly rare during these communication attempts in non-lucid REM sleep, as well as during periods when two-way communication was not attempted, lends additional credence to our position that correct signals were not spurious but rather reflect successful cases of communication during lucid dreaming. For additional details, see STAR methods.

## DISCUSSION

We have presented four independent examples in Figures 2, 3, 4, and 5 of successful dialogue between experimenters and dreamers. Each team used somewhat different procedures and yet all findings converged to establish real-time dialogue between experimenters and dreamers during REM sleep. Our findings, as summarized in Tables 1 and 2, refute the common belief that it is pointless to try to communicate with people who are asleep to gain knowledge about their dreams, and the assumption that they cannot respond in any meaningful way while remaining asleep. On the contrary, the collection of results described here constitutes proof of concept of two-way communication during sleep, and thus opens the door to a new approach for scientific exploration of the dream state.

Before accepting these findings, it is important to thoroughly evaluate the evidence, starting with the question of whether these episodes occurred entirely during REM sleep. In other words, to what extent can we confirm that participants were asleep when the presumptive communication took place? Our approach here was to rely on standard criteria from contemporary sleep research for scoring sleep physiology,^24^ which substantiated the REM sleep state during these examples of two-way communication. We also relied on a thorough evaluation of the data by three sleep experts who provided unbiased scoring of the polysomnographic data to confirm intervals of REM sleep using standard criteria.

Nevertheless, the conventional physiological criteria, widely used and accepted in contemporary research and clinical contexts, may be improved in the future, changing how sleep is defined. Additionally, one might invoke the possibility that some parts of the brain can be in REM sleep while others are not. Some aspects of REM sleep physiology resemble both the waking state and stage N1 (the first stage of non-REM sleep), which is when hypnagogic hallucinations can be observed. Speculatively, three stages—REM, N1, and waking—could be present at the same time in different brain areas. Although sleep researchers have conjectured about this notion of local sleep,^25,26^ hybrid sleep stages have yet to be introduced into standard analyses of sleep physiology. Analytic techniques that capture the detailed spectral composition of sleep signals (e.g., Prerau et al.^27^) may spur the development of fine-grained categorization schemes for sleep stages. Indeed, the present methods and results may be helpful for future explorations of such possibilities.

One limitation of the procedures we used is that they do not always produce interactive dreaming. In some cases, sensory gating or competition from endogenous events^28^ may prevent participants from perceiving the stimuli and their meaning, or the meaning might be distorted. Alternatively, stimuli may produce arousal from sleep, or people may wake up while attempting eye signals. These problems were prevalent in the course of the present research, and yet we were able to avoid these pitfalls on multiple occasions. Other investigators have explored pharmaceutical approaches to stabilize REM sleep.^29,30^ We encourage further efforts that may produce additional strategies to optimize procedures. Interestingly, lucidity can be tenuous, in that individuals can transition from lucid dreaming one moment to believing that the experience is a waking experience the next, and maybe back again. The present studies did not allow us to formally compare the likelihood of two-way communication during lucid dreams versus non-lucid dreams, because our goal was to communicate during lucid dreams. Addressing this issue is an exciting challenge for future research.

Prior research set the stage for interactive dreaming in important ways, but here we take a leap beyond what has been documented before. We demonstrate that it is possible to perceive and respond to complex questions during sleep, and that dreamers can correctly respond to these queries without knowing what would be asked in advance. Correct responses in our results were ascertained through visual inspection by the experimenters, subsequently verified when we subjected the data to independent appraisal to assure that signals were judged in an unbiased way. Our procedures for two-way communication differ from the procedures in two studies in which an expert lucid dreamer knew precisely what stimuli would be presented and how to respond to them.^31,32^ These prior studies documented minimal communication using only simple tones and shocks. Likewise, in a study not concerning lucid dreaming, Mazza and colleagues^33^ presented 20 nociceptive stimuli (5-ms laser pulses to the hand that produced painful heat sensations during wake) to an epileptic patient during REM sleep, and she responded to 11 of them with a finger response as she had done previously while awake. There was no indication that these stimuli were incorporated into a dream and no recollection of the stimulation after awakening. Should the transfer of even a small amount of information be considered as a minimal form of communication? Also, does it matter if the response is not volitional (e.g., tap to the patella followed by a reflexive response)? Communication can perhaps take many forms, but dialogue implies a richer sense of communication. When the form of the interchange is specified in advance, a response from a dreamer may primarily reflect their expectations and pre-existing habits, precluding conclusions about communicative capabilities during a dream. In our examples of two-way communication, substantial information not known in advance was transferred in both directions between two individuals, as in a conversation. The present results (acknowledging preliminary non-peer-reviewed reports^34,35^) thus represent an advance in demonstrating two-way communication of novel information that was not pre-determined. Furthermore, given the complexity and variety of the questions posed, the results obtained during sleep in combination with the post-sleep dream reports suggest that the signals produced from within a dream were volitional answers.

Notably, we infer that our participants demonstrated preserved cognitive abilities while asleep in several respects. They were able to remember pre-sleep instructions on how to respond, and then apply them during sleep to novel, externally presented queries. They engaged working memory operations to perform mathematical computations and accessed autobiographical memories about their waking life. There may be ways in which dreamers are limited in their cognitive abilities, perhaps due to dorsolateral prefrontal deactivation during REM sleep.^36^ Indeed, people typically lack the analytic ability to recognize that they are dreaming. Yet here we provide evidence that many advanced cognitive abilities can be engaged in a dream. Of course, dream reports per se suggest that a wealth of cognitive activity is engaged during sleep. However, inferring cognitive abilities from a dream report alone requires accepting that dream reports are veridical, which can be doubtful. Thus, inferring cognitive abilities from responses made via real-time interrogation by an experimenter belongs in a different category. Interactive dreaming provides a novel method to compare cognitive abilities across states, as tasks previously administered only in waking participants, such as working memory tasks, can now be administered during REM sleep.

The standard view has long been that sleeping individuals are oblivious to the world around them, their senses effectively shut down to allow in only the strongest stimuli, making comprehension and meaningful dialogue impossible—this view must be updated. The integration of external stimuli into dreams has been documented at least as far back as Aristotle.^37–39^ The data presented here underscore how meaning delivered during sleep can influence dream content. Sometimes stimuli were perceived as coming from outside the dream, but other times the stimuli emanated from elements of the dream, contextualized in a way that made sense in relation to ongoing dream content. Further studies are needed to determine what factors influence how stimuli are perceived within a dream, and interactive dreaming is uniquely positioned for addressing these questions.

Our results also document robust examples of sleep learning.^40^ For example, when the participant awoke after the procedure shown in Figure 2 and reported that he had been asked to compute the answer to a simple subtraction problem, he was displaying information learned while he was asleep. He acquired novel and specific knowledge in the form of an episode with the spoken question, what is 8 minus 6?—recollective knowledge of a declarative memory that he recalled verbatim. This prime example of explicit recollection stands in contrast to previous reports of new learning in sleeping individuals, as the verified acquisition of new information has been limited to conditioning and basic perceptual learning.^21,41^

Procedures for interactive dreaming such as those documented here could be adapted to facilitate many potential applications. That is, dreams could be curated in accordance with an individual’s objectives, such as to practice a musical or athletic skill. Prior studies suggest that dreaming about facts or skills one is trying to learn can correlate with enhanced performance.^42,43^ Dreams can also provide a unique opportunity to lessen the impact of emotional trauma.^44,45^ Thus, cues could be devised in advance to influence dream content,^46^ or be modified based on the dreamer’s preferences signaled during a dream. In addition, interactive dreaming could also be used to solve problems and promote creativity—the next moonshot ideas could be produced with an interactive method that can combine the creative advantages of dreaming with the logical advantages of wake. Artists and writers might also gain inspiration from sleep communication.^47^

The scientific investigation of dreaming, and of sleep more generally, could be beneficially explored using interactive dreaming. Specific cognitive and perceptual tasks could be assigned with instructions presented via softly spoken words, opening up a new frontier of research. Indeed, such an approach would overcome the traditional difficulties preventing a rigorous scientific investigation of dream functions, namely the lack of access and control over dream timing and content. If we can query people about the content of their dreams, we can then recommend changes in dream content, and monitor concurrent brain activity. A window into events that occur in the course of a dream could also be used to quantify the extent to which dream reports are distorted upon waking.^48^ In addition, novel approaches to promote health and well-being could be explored.^49^ Neural decoding methods^2–5^ could also be applied in various creative ways. Based on the current results, we suggest that future studies might consider shorter intervals for sleep staging (to avoid cases where part of an interval is REM with two-way communication, followed by an awakening, requiring the entire interval be designated as wake, as occurred sometimes in our studies). Using bidirectional communication with dreamers, we could address many unanswered questions about sleepers’ phenomenological experiences (e.g., probing time perception across sleep cycles by asking how much time has elapsed since the last query, and examining how dream experiences vary across stages). Experiments from many corners of cognitive neuroscience can be modified and applied to interactive dreaming, perhaps opening up new ways to address fundamental questions about consciousness.

In summary, we demonstrated that two-way communication with dreamers is a replicable phenomenon across different participant populations, lucid-dream-induction techniques, and communication paradigms. These efforts culminated in what we term “interactive dreaming.” We’ve long known that cognition and consciousness are not shut off during sleep, but our results now broaden the opportunities for empirically peering inside the sleeping mind. The advent of interactive dreaming—with new opportunities for gaining real-time information about dreaming, and for modifying the course of a dream—could usher in a new era of investigations into sleep and into the enigmatic cognitive dimensions of sleep.

## STAR⋆METHODS

### RESOURCE AVAILABILITY

#### Lead Contact

Requests for further information and resources can be directed to the lead contact, Ken Paller (kap@northwestern.edu). Individual groups will have responsibility for their own resources.

#### Materials Availability

This study did not generate reagents.

#### Data and Code Availability

Publicly available software used for analyses is listed in the Key Resources Table. Data and code used in this study will be shared upon request from a qualified investigator at an academic institution, subject to negotiation and decision of a university review and data-use agreement process.

### EXPERIMENTAL MODEL AND SUBJECT DETAILS

#### Participants

Thirty-six adults participated in the study. Demographic details are provided below separately for each research group. Experiments from each group were approved by ethics review at the researchers’ respective institutions. All participants gave informed consent.

### METHOD DETAILS

#### Methods Common to All Research Groups

##### Sleep scoring

Two-way communication was generally attempted during a period of REM sleep as assessed online. Following data collection, each group scored their own polysomnographic data following standard procedures. Then, three certified sleep scorers (medical doctors with degrees in sleep and pathology, Diplôme Inter-Universitaire Le sommeil et sa pathologie), who were blind to initial sleep scoring, were recruited to conduct independent sleep scoring.^24^ Sleep scorers were also blind to which periods contained two-way communication attempts. They scored a sample of 30-s periods, including all two-way communication attempts, the periods immediately before and after communication attempts, some unambiguous REM sleep periods without two-way communication attempts, and wake periods with and without LRLR practice.

Each group provided a file with their sleep data organized by subject and session number, indicating the epoch number of each 30-s period so that scorers would know which pages were continuous. The sleep data included at least one frontal, central, and occipital EEG channel (except in rare cases where multiple electrodes failed and scoring was done with remaining available electrodes), two EOG channels (electro-encephalography), and one chin EMG (electromyography), and were filtered in the same way (0.3 to 15 Hz for EEG and EOG data, 10 to 100 Hz for EMG data, and a calibration marker to indicate an amplitude of 100 μV). For the French data, corrugator and zygomatic facial EMG channels were not shown. Certified scorers scored a total of 1652 periods (214 for USA group; 1290 for German group; 75 for French group; 73 for Dutch group). A subset of 850 periods included two-way communication attempts (93 for USA group; 685 for German group; 35 for French group; 37 for Dutch group). Inter-scorer agreement was high (Fleiss’ kappa = 0.71 for all data; Fleiss’ kappa = 0.70 for two-way communication periods).

To establish whether two-way communication attempts were successful, we focused on attempts during periods that were scored as REM sleep by at least two of the three independent sleep scorers and belonged in sessions with signal-verified lucid dreaming. Among these trials, 80.4% (127/158) were scored as REM sleep by all three experts. A summary of the data that we used for this analysis is provided in Table 1. A score of REM sleep was unanimous for the epochs shown in Figures 2 and 5, and 2 of 3 blind scorers agreed on REM sleep for the epochs shown in Figures 3 and 4.

##### Signal scoring

For two-way communication attempts, each group independently rated whether there was a signaled response and, if so, quantified eye movements or facial muscular contractions. Then, three independent raters who were completely naive to the number of eye signals expected or the number and type of muscle contractions were recruited to evaluate all data from each group. These individuals were not highly familiar with lucid dreaming and were blind to the initial score. Each group provided a file with their signal data organized by subject and session number, including all channels (USA) or only the signal of interest (French, Dutch, and German data). An arrow indicated the beginning of each communication attempt. Raters indicated when they saw an eye signal, a zygomatic contraction, or a corrugator contraction. They also indicated whether they were certain, had moderate confidence, or low confidence in each case, and we only counted ratings that were made with high confidence. For the EOG data, results across scorers varied, in part because the individuals did not adopt the same criteria for what constituted a signal, which was likely a downside of the fact that they were not familiar with lucid dreaming. Accordingly, the analysis was possibly too stringent and may have omitted some valid signals.

We classified each trial into one of the following four categories: correct response, incorrect response, ambiguous response, or no response. For this categorization, we included the original rating of the signal along with the three independent ratings. For US, Dutch, and German eye-movement data, trials were considered as:

- Correct: if 3 out of the 4 raters agreed that the count matched the expected response (example in Figure S1A)
- Incorrect: if 3 out of the 4 raters agreed that the count was other than the expected count (example in Figure S1B)
- Ambiguous Response: if 3 out of the 4 raters agreed there was a response but they did not agree on the count (example in Figure S2A), or if 2 raters thought there was no response (example in Figure S2B)
- No Response: if 3 out of 4 raters agreed that no signal was given (example in Figure S3).

For the French data, we evaluated responses in the tactile task following the same rules as above (example in Figure S4). Our procedure was slightly different for other tasks (yes/no questions, tone discrimination, and semantic-discrimination task) because participants had to respond using one of the two response channels (corrugator or zygomatic muscles) and the signal consisted of two contractions. In those cases, trials were considered correct when the majority of raters agreed that the expected muscle was contracted twice, incorrect when the majority of raters agreed that the wrong muscle was contracted twice, and as no response when the majority of scorers agreed there were no contractions. When the count of the expected muscle was different than two, or if raters did not agree on the contraction count, we considered the trial as an ambiguous response.

To evaluate whether the eye movements that we considered as responses during REM sleep could have happened by mere chance, we analyzed trials with two-way communication attempts that occurred in non-lucid REM sleep (i.e., when there was no signal of lucidity during sleep and no subsequent dream report of lucidity). Otherwise, we followed the same analysis procedure as described above. In total, we analyzed eye-movement responses on 379 trials (German, n = 347; USA, n = 32; Dutch, n = 0). We found few eye signals in these control conditions, with 1 correct response, 1 incorrect response, 11 ambiguous responses, and no response on 366 trials (Fischer exact test, p < 0.001, number of responses higher during two-way communication attempts during lucid REM sleep versus non-lucid REM sleep). We also included another method for assessing chance-level performance focused on periods of lucid dreaming. For facial contractions (French data), we analyzed data from the semantic-discrimination task during 1-min periods without any stimulus presentation. We added 28 markers every 10 s to indicate the beginning of a possible response interval, approximating what happened during the task. Raters were not informed about which trials were control trials and which were two-way communication attempts. We observed no facial muscle contractions (correct or incorrect) during sham trials (Fischer exact test, p < 0.001, number of responses higher during lucid REM sleep with two-way communication attempts compared to without TWC attempts). In sum, the first analysis showed that correct signals seldom occurred when there was no prior signal of lucidity during sleep and no subsequent dream report of lucidity. However, this estimate of chance-level accuracy (1 out of 379) could be questioned if random responding happens preferentially in lucid dreams. In the second analysis, chance-level accuracy during lucid dreaming was still low (0 out of 28). Admittedly, this estimate was derived from a single lucid dreamer. However, an additional argument against the notion that the correct answers we observed were merely random responding is derived from the data in Table 2. Across our studies, the number of correct answers was 29. These correct answers were from six different participants, all experiencing signal-verified lucid dreams during REM sleep. Importantly, the number of correct answers was much greater than the number of incorrect answers (29 versus 5), which is inconsistent with the possibility that random responses were produced here. Across the math problems, there were four or more possible answers, meaning that less than one out of four would be answered correctly with a random response (i.e., there should be over three times as many incorrect responses as correct responses). Thus, we can be confident that successful cases of communication during REM sleep did not happen merely by chance.

It is interesting to note that a few participants responded to math problems outside of signal-verified lucid dreams (Figure 5). Although here we focused on trials of two-way communication that occurred after lucidity was confirmed via standard eye signals, it may be interesting to consider whether responding to a question during sleep could constitute a form of signal-verification. In Figure 5, the dreamer did not perform lucid signals prior to the two-way communication attempt, but subsequently reported that they were lucid while responding (although note that this was their second nap in the lab, and they were able to perform lucid signals in the initial baseline nap). In another example from the German group, however, a math problem (5 minus 2) was correctly answered during REM sleep without either signal-verification or a dream report of lucidity. Indeed, the dreamer reported, “I am in the bed in the sleep lab, and I know that my task is to solve math problems, which are delivered to me with blinking lights or beeping tones. I realize at some point that the lamp has been beeping for quite some time [the actual lamp in the sleep lab does not beep]. I concentrate on solving the math problem. The answer is ‘3’ and I report it with the eye movement. I am not aware that I am dreaming. I think ‘6 minus 3’ was the math problem, but I am not sure if this was really the math problem. I can only confidently remember the solution.” While these cases were not considered signal-verified lucid dreams and therefore not included in the total count of two-way communication trials here, they raise the interesting issue of how signal-verification should be defined moving forward.

#### Procedures for Group in the USA

##### Participants

Twenty-two participants (15 female, age range 18–33 years, M = 21.1 ± 4.3 years) who claimed to remember at least one dream per week were recruited by word of mouth, online forum, and the Northwestern University Psychology Department participant pool. They each participated in one or more nap sessions, which amounted to 27 nap sessions in total.

##### Procedure

Participants visited the laboratory at Northwestern University at approximately their normal wake time and received guidance on identifying lucid dreams and instructions for the experiment for about 40 min during preparations for polysomnographic recordings, including EEG, EMG, and EOG, using a Neuroscan SynAmps system. Participants were instructed to signal with a prearranged number of LR eye movements if they became lucid in a dream.

Next, participants practiced making ocular signals and responding to questions using combinations of LR signals. Subsequently, participants completed the Targeted Lucidity Reactivation (TLR) procedure while lying in bed. This procedure was derived from the procedure developed by Carr and colleagues.^50^ A method of reality checking to induce lucid dreaming was paired with sensory stimulation and accelerated in a single session immediately before sleep, and then cues were presented again during REM sleep. In this procedure, participants were trained to associate a novel cue sound with a lucid state of mind during wake. The sound consisted of three pure-tone beeps increasing in pitch (400, 600, and 800 Hz) at approximately 40–45 dB SPL and lasting approximately 650 ms. For one participant, the pure-tone beeps had previously been associated with a different task in an unrelated study. Thus, for this participant, a 1000-ms violin sound and low-intensity flashing-red LED lights were used as cues. All participants were informed that this cue would be given during sleep to help promote a lucid dream. Over the next 15 min, the TLR sound was played up to 15 times. The first 4 times, it was followed by verbal guidance to enter a lucid state as follows. “As you notice the signal, you become lucid. Bring your attention to your thoughts and notice where your mind has wandered…[pause] Now observe your body, sensations, and feelings…[pause] Observe your breathing…[pause] Remain lucid, critically aware, and notice how aspects of this experience are in any way different from your normal waking experience.”

Participants often fell asleep before all 15 TLR cue presentations were completed. Standard polysomnographic methods were used to determine sleep state. Once participants entered REM sleep, TLR cues were presented again, at about 30-s intervals, as long as REM sleep remained stable. After participants responded to a cue with a lucid eye signal, or after approximately 10 cues were presented without response, we began the math problem portion of the experiment.

We devised the following task to engage auditory perception of math problems, working memory, and the ability to express the correct answer. We used simple addition and subtraction problems that could each be answered by a number between 1 and 4 (LR = 1, LRLR = 2, LRLRLR = 3, LRLRLRLR = 4), or between 1 and 6 for the first 5 participants.

In the physiological recording in Figure 2, the participant reported an experience consistent with a lucid dream, sleep paralysis, or a combination of the two. Both states involve similar neurophysiology and can be characterized as dissociated REM sleep phenomena.^51^ Whereas sleep paralysis can also occur when muscle atonia is accompanied by alpha, which is often associated with arousal,^52^ in this example the participant showed very little alpha. Further, while sleep paralysis is sometimes described as occurring between REM and wake, in this example the participant responded to the math problems shown in Figure 2, but not the two presented subsequently. We would expect that if the participant was communicating from a state between REM and wakefulness, he would have answered these final math problems also.

#### Procedures for Group in Germany

##### Participants

Ten healthy participants (4 female, age range 21–40 years, M = 26.8 ± 6.3 years) were recruited from Germany via forum posts in lucid dreaming internet forums and via the local university student mailing lists. They were all experienced lucid dreamers who claimed to have had at least one lucid dream per week and at least 35 lucid dreams before the study (130 ± 156.5).

##### Procedure

Before coming to the sleep laboratory, participants underwent an internet-based training program at home to learn how to decode Morse-coded messages containing math problems (i.e., the numbers 0 to 9 and the letters “P” and “M” for “Plus” and “Minus”). Participants opened a website programmed in HTML5 and Javascript. This website provided as many training examples as the participant wanted, both for visual stimuli (i.e., screen flashing in red and black, detectable through closed eyes in a dark room) and for acoustic stimuli (1000-Hz pure tones). For example, the math problem “3 plus 6” would translate to 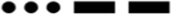, 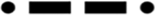, 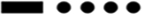, with dots representing short 300-ms flashes/tones and dashes representing long 900-ms flashes/tones. The participants could adjust the speed as desired. They were asked to train until they were sure to be able to decode visually and acoustically Morse-coded math problems literally during sleep. There was a 300-ms pause after each stimulus and a 3000-ms pause between each numeral or operator of the math problem.

Furthermore, participants were instructed to give answers to the math problems using eye movements. There was a 20-s pause for answering after each math problem. An eye movement from the center of the visual field to the left and back to the center (“left”) corresponds to a Morse code dot, and the reverse eye movement from center to right and back to center (“right”) corresponds to a Morse code dash. For example, the eye-movement sequence of “right-right-right-right-left” would translate to 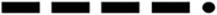 to produce the answer “9” and would last about 5 s.

After arriving at the sleep laboratory at Osnabrück University, participants were asked to demonstrate their Morse decoding skills for both flashing and beeping stimuli during wakefulness, in order to make sure that they were proficient, which was the case for all participants. Moreover, EOG was used to display their eye movements in real-time on a computer screen. Participants were next asked to practice Morse-coded eye movements, which had to be clearly visible in the EOG. Five participants were not able to do so, including the participant whose results are in Figure 3. These participants were asked to use a simpler answering procedure by moving their eyes from left to right and back a number of times to indicate as their answers (i.e., the answer “4” would be given by four LR eye movements).

The math problems were selected so that operands as well as solutions ranged from 0 to 9. For participants who used the simpler answering procedure, solutions ranged from 2 to 5. Problems were generated randomly by a computer algorithm while the participant was asleep, such that both experimenter and participant could not know which problems to expect.

Participants spent two or three nights in the sleep laboratory. They underwent polysomnographic recordings using a Neuroscan Model 5083 SynAmps system, including 19 EEG channels from the 10–20 system, horizontal and vertical EOG, and chin EMG. Impedance was below 5 kΩ at the beginning of the night. Data were sampled at 500 Hz.

Participants were sent to bed at around 11 PM. Then, 4.5 h after sleep onset, the experimenter waited for the next REM sleep period to occur. After 10 min of REM sleep, the participant was woken up. Next, the participant stayed awake for 45 min and was requested to practice solving visual and acoustic math problems. The participant was also asked to conduct lucidity-promoting exercises of his/her own choice or the exercise of identifying dream signs in dream reports with an autosuggestion technique. Before the participant was sent to bed again, the signal quality of the EEG, EOG, and EMG electrodes was improved if necessary.

During each of the following REM sleep periods, first the stimulus condition (acoustic or visual) was randomly selected. Next, after 5 min of stable REM sleep, stimuli consisting of Morse-coded math problems were presented to the sleeping participant. Acoustic stimuli (pure tones at either 470 Hz or 600 Hz) were delivered via computer speakers. Visual stimulation was delivered using an LED strip, which brightened the sleep chamber in red or green colors. The tone frequencies and LED colors were used in alternating order such that the participant could more easily identify the beginning of a new math problem. Stimulus intensity was gradually increased for each new problem until either the participant responded with eye signals, had an arousal, or went into non-REM sleep, in which case stimulation was stopped. Stimuli were generated and presented using custom Python scripts.

Participants were instructed to move their eyes three times from left to right when they realized that they were dreaming (“lucidity signal”). If there was no stimulation ongoing already, stimulation was started immediately following the lucidity signal. If 2 min elapsed after the last eye signal (lucidity signal or math answer) without an awakening, the participant was awoken and asked to complete a written dream report as well as a questionnaire about the sleep communication. Recordings were stopped in the morning when the participant stated not being able to sleep anymore. The procedure is summarized in Figure S5.

#### Procedures for Group in France

##### Participants

To test the possibility of communicating with a dreamer, we used sensory stimulation in one experienced lucid dreamer with narcolepsy. We have recently shown that patients with narcolepsy had many advantages for lucid dreaming research. First, 78% of these patients were lucid dreamers^53,54^ achieving an average of 8 lucid dreams per month without any specific training. Furthermore, narcolepsy, by definition, is characterized by excessive daytime sleepiness and abnormal transitions between wakefulness and REM sleep including rapid entry into REM sleep.^55^ These unique features allow collection of lucid REM sleep episodes in only a few daytime naps in a sleep lab.^56^ Of note, the overall structure of sleep is conserved in narcolepsy. Although some EEG features have been identified during narcoleptics’ REM sleep (e.g., increased alpha power), none of these features were sufficient to differentiate REM sleep in narcolepsy from REM sleep in healthy controls.^57^

We recruited patient AC to participate in a two-way communication study. Patient AC is a 20-year-old male diagnosed with narcolepsy (type 1) at the age of 16. He developed extraordinary lucid dreaming abilities, with a remarkable ease for controlling dream scenarios. He reports a daily average of 4 lucid dreams, including daytime naps and nighttime sleep.

##### Procedure

Patient AC visited the lab for daytime naps. We tested his ability to respond to stimuli from different sensory modalities (auditory, tactile, and visual, see Figure S6). To measure AC’s responses to stimulation, we used three signals based on the EMG activity of facial muscles: the codes were brief, successive contractions of either the corrugator muscle, the zygomatic muscle, or an alternation of these two facial muscles. Even if the sleeper is paralyzed during REM sleep, isometric muscular activations or muscle twitches are often observed during this stage.^58^ Patient AC considered these facial signals easier and quicker to produce, less arousing, and less disturbing of an ongoing dream than the left-right eye movements.

In the first session, AC had to indicate lucidity with the following sequence: LRLR, corrugator contraction, zygomatic contraction, LRLR. This sequence was used to validate facial EMG codes during lucid dreams. Then, patient AC had to either contract the corrugator or zygomatic muscles to: i) count the number of taps applied on his right hand, ii) discriminate between low- and high-pitch auditory tones, iii) answer yes/no questions, or iv) discriminate between visual light changes. AC was told about the different tasks that he would have to perform (and the corresponding muscular signals), but not of the order of the different stimulation modalities. Thus, he was not able to predict which and when the stimulation would be applied.

In the second session several months later, AC had two naps. This time, we presented auditory stimuli repetitively during 10 blocks. The stimuli were French words (meaning *up*, *down*, and *mixed*) and were pronounced by a female voice through speakers using Psychtoolbox extension for MATLAB (MathWorks). Each block included 6 auditory stimuli (randomly chosen), presented every 10 s with a jitter. The blocks were separated by a 1-min period during which only white noise was presented (OFF period without stimulation). The stimulation session lasted for 20 min. Stimulation started 1 min after the beginning of the nap, when the patient was still awake. AC was instructed to perform a semantic task whenever he was in a lucid dream and heard a stimulus: he had to perform two zygomatic contractions if he heard the word *up*, two corrugator contractions if he heard the word *down*, and one corrugator contraction followed by one zygomatic contraction if he heard the word *mixed*. AC reported being lucid during the first nap. In this nap, 39 words were presented during REM sleep. Results are shown in Table 2. AC did not reach REM sleep during the second nap and did not report any lucid dreams.

#### Procedures for Group in the Netherlands

##### Participants

Healthy volunteers (N = 37, 23 female, age range 19–37 years, M = 23.2 ± 4.2) were recruited through the Donders Institute for Brain and Cognition SONA system. To verify their eligibility, they were assessed through questionnaires for general health, general sleep quality (Pittsburgh Sleep Quality Index–PSQI^59^), dream recall and lucidity frequency (Mannheim Dream questionnaire–MADRE^60^); Attitude Toward Dreams questionnaire^61^), altered nocturnal behaviors (Munich Parasomnias Screening–MUPS^62^), chronotype (Munich Chronotype Questionnaire–MCTQ^63^), and mental imagery (Psi-Q^64^). Moreover, we included participants who declared a dream recall frequency of at least 3 times per week and had the experience of at least one lucid dream in their lives. We gave priority to participants with a consistent sleep schedule in the month before the assessment and absence of sleep disturbances. From this sample, N = 13 participants (10 female, age range 19–37 years, M = 23.9 ± 5.22) were suitable for this study.

##### Procedure

The experimental procedure is graphically represented in Figure S7. The lucid dream induction procedure is similar to that used by the USA group, drawing on the same recently established targeted lucidity reactivation (TLR) procedure to induce lucidity during morning naps via acoustic and visual cues.^50^ Participants who succeeded in reaching dream lucidity during a first baseline nap were tested for two additional naps. They kept a sleep- and dream-log for 7 days before the first experimental session, which measured their dream recall and lucid dreaming frequency (Dream Lucidity Questionnaire–DLQ).^65^ Dreams were recorded each morning through an audio recorder to promote compliance in maintaining a regular sleep schedule.

Once participants arrived at the EEG lab at 7:00 a.m., we explained the experiment and wired them up to 128-channel high-density EEG (BrainAmp MR Plus, Brain Products). Before the nap, they underwent a 15-min TLR training session to increase lucid dreaming propensity, as described in the USA procedure section, except that the TLR cues for all participants included both auditory (beeping tones) and visual (blinking lights) cues. Auditory cues were presented on a background of white noise, set at a maximum of 45 dBA (whisper-like). TLR cues were administered at 1-min intervals during the mindfulness training. Participants were further instructed on how to signal that they were lucid dreaming while sleeping, using a sequence of two left-right rapid eye movements during REM sleep.^7,8^ Finally, participants had the opportunity to sleep for at least 90 min.

TLR cues were administered again after the first 30–s epoch of REM sleep, one cue every 10–15 s. Cues were paused if the EEG showed an arousal and resumed at REM-sleep onset. Up to 10 cues were presented during each REM sleep period. Participants were instructed to perform the eye signals once lucid, and to keep signaling the state of lucidity every 10 s, while taking control of the oneiric scene. The experimenter woke up participants after they stopped giving eye signals. Then, the experimenter collected dream report and lucidity measures. Levels of lucidity, awareness, control of the oneiric scene were rated on Likert scales ranging from 1 to 9. The Dream and Lucidity Questionnaire was used to evaluate different features of awareness, control and remembrance, and has 12 items scored on a 5-point scale ranging from 0 to 4.^65^

Participants who successfully reached lucidity during the first nap (baseline) were tested for two additional naps with a dream communication procedure. The procedure for inducing lucidity was the same as the first session. In addition, participants practiced answering math problems with eye movements before going to sleep. Problems were presented with softly spoken words, and participants were instructed to look left-right once for each number in their response. In case participants perceived only part of the math problem in the dream (e.g., 3 instead of 3-1), they were instructed to signal the number they heard.

During the sleep portion, when a lucid eye signal was recognized online by the experimenter, the math problems were administered every 10–15 s and recorded so that the verbal math problems could be matched to the eye movements measured with EOG. If no eye signal was detected on the EOG, the experimenter kept administering math problems until REM sleep ended or the dreamer woke up. When participants woke up, they reported their dream, whether they heard any of the stimuli during sleep, and whether they remembered solving math problems in the dream.

## Supplementary Material

1

## Figures and Tables

**Table 1.** Summary of data collection from each team

Team	Participants	Lucid dreaming method	Tasks	Output signals	Sessions with TWC attempts	Sessions with REM sleep	Sessions with SVLD	Trials with TWC attempts
USA	people who remembered ≥1 dream/week (n = 22)	targeted lucidity reactivation	spoken math questions	eye movements	16	12	6	31
Germany	experienced lucid dreamers with ≥35 lucid dreams total (n = 10)	wake-back-to-bed method	math questions indicated by tones and lights	eye movements	60	40	5	54
France	an experienced lucid dreamer with narcolepsy (n = 1)	spontaneous lucid dreaming	spoken yes/no questions; discrimination of tactile, speech, and light stimuli	facial muscle contractions	2	2	2	65
the Netherlands	people who remembered ≥3 dreams/week with ≥1 lucid dream (n = 3)	targeted lucidity reactivation	spoken math questions	eye movements	4	3	2	8
Totals	N = 36				82	57/82	15/57	158

TWC, two-way communication; SVLD, signal-verified lucid dreaming. Targeted lucidity reactivation entails training with sensory stimulation prior to sleep, followed by sensory stimulation during sleep. The wake-back-to-bed method entails arousal from sleep for 15–60 min followed by the intention to lucid dream upon returning to sleep. A trial corresponds to a single two-way communication attempt, as in delivering a math question. Our analysis was restricted to trials that occurred during REM sleep with SVLD.

**Table 2.** Observed responses during two-way communication attempts in REM sleep periods with signal-verified lucid dreaming

Team	Task	Total trials	Correct responses	Incorrect responses	Ambiguous responses	No responses
USA	math problems	31	6	1	5	19
Germany	math problems in Morse code and Morse-code eye movements	4	0	0	0	4
	math problems in Morse code and LR eye movements	50	1	2	2	45
France	counting (tactile)	13	7	2	2	2
	sound discrimination	4	0	0	2	2
	light discrimination	4	0	0	0	4
	semantic discrimination	39	12	0	14	13
	yes/no questions	5	2	0	2	1
the Netherlands	math problems	8	1	0	1	6
Total		158	29 (18.4%)	5 (3.2%)	28 (17.7%)	96 (60.8%)

All trials were scored as REM sleep by at least two of three expert sleep scorers. Three additional raters, while blind to condition, rated the number of eye movements or muscle contractions after each two-way communication attempt. An experimenter was included as a fourth rater. The identity of each signal, or the absence of a signal, was determined based on consensus (at least three of the four raters). If there was no such consensus, the signal was counted as an ambiguous response. If a signal matched the correct answer, it was considered a correct response. If a signal was not the correct answer, it was considered an incorrect response.

**Table:** KEY RESOURCES TABLE

REAGENT or RESOURCE	SOURCE	IDENTIFIER
Software and Algorithms		
MATLAB 2020b	RRID: SCR_001622	https://www.mathworks.com/products/matlab.html
Python 2.7	RRID: SCR_008394	https://python.org
Javascript	Javascript	https://www.javascript.com/
Psychtoolbox for MATLAB	RRID: SCR_002881	https://www.mathworks.com/matlabcentral/fileexchange/76411-psychtoolbox-3

## Highlights

- Dream reports given after people awaken are often fragmentary and distorted
- Our methods allow for two-way communication with individuals during a lucid dream
- For a proof-of-concept demonstration, we presented math problems and yes-no questions
- Dreamers answered in real time with volitional eye movements or facial muscle signals
