# Supplementary material for: Real-time dialogue between experimenters and dreamers during REM sleep
Source: Curr Biol. Author manuscript; Available in PMC 2021 May 28. (PMC8162929; doi:10.1016/j.cub.2021.01.026)
Supplement: 1 [file NIHMS1704833-supplement-1.pdf]

Current Biology, Volume *31*

Supplemental Information

Real-time dialogue between experimenters

and dreamers during REM sleep

Karen R. Konkoly, Kristoffer Appel, Emma Chabani, Anastasia Mangiaruga, Jarrod Gott, Remington Mallett, Bruce Caughran, Sarah Witkowski, Nathan W. Whitmore, Christopher Y. Mazurek, Jonathan B. Berent, Frederik D. Weber, Başak Türker, Smaranda Leu-Semenescu, Jean-Baptiste Maranci, Gordon Pipa, Isabelle Arnulf, Delphine Oudiette, Martin Dresler, and Ken A. Paller

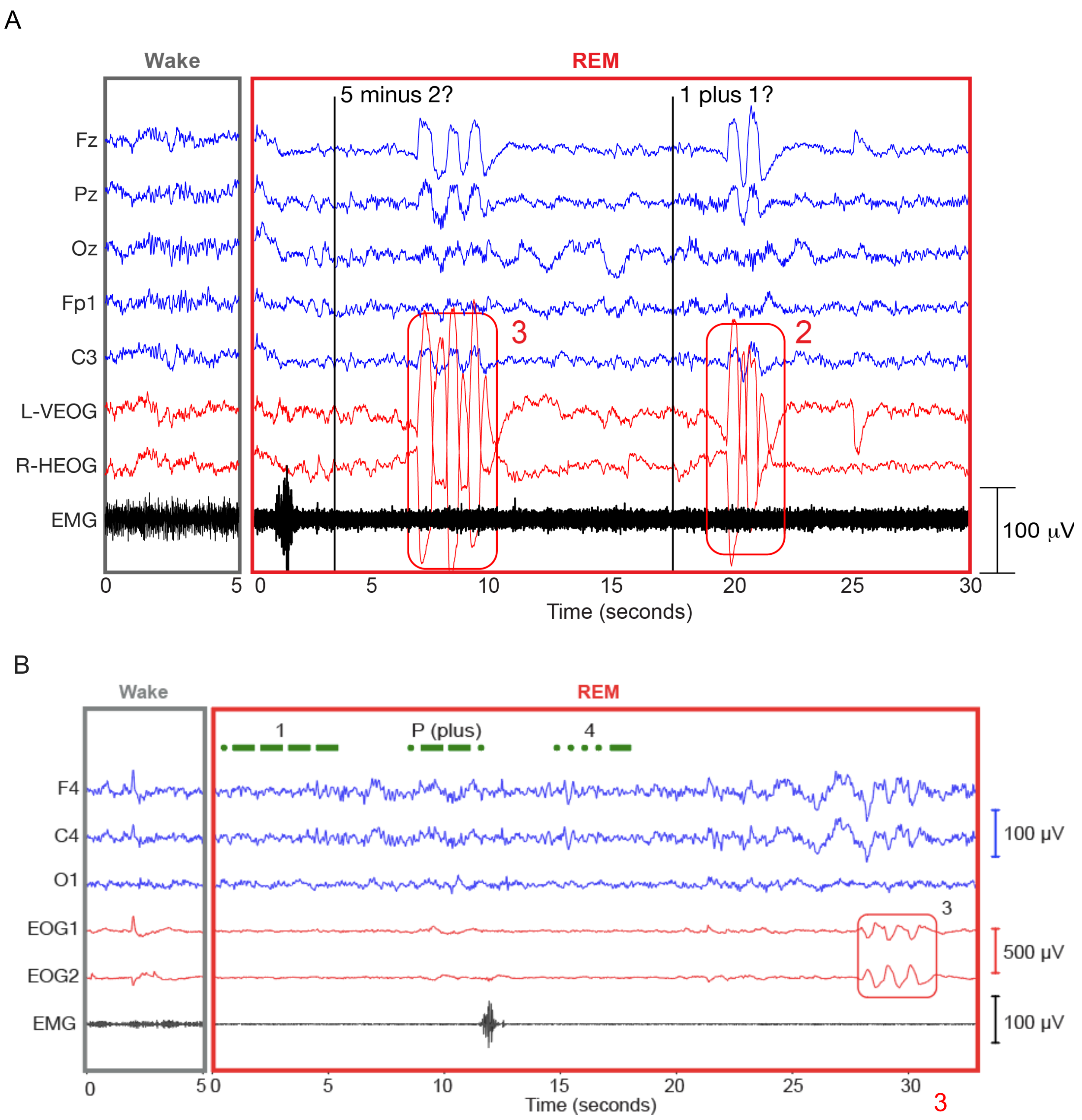

Figure S1. Correct and Incorrect Responses, Related to STAR Methods.

A: An example of two math trials categorized as "correct." Upon awakening, the dreamer reported, "I don't really remember anything visually. I was singing a song, or like a song was going through my head. It was the Barber of Seville." The experimenter asked if they heard any sounds or math problems while sleeping, what the problems were, and how they answered. The participant said, "I remember 5 minus 2. I don't remember the other one. I know there was another one... I gave 3 for 5 minus 2."

B. An example of a math trial categorized as "incorrect." Scorers agreed that the response was 3, but the correct response was 5. Upon awakening, the dreamer reported, "I do a nose reality check several times and am lucid. I give LRLRLR. Kristoffer asks me via intercom whether this was an eye signal. I give LRLRLR. A researcher enters the room and gives me a high five, because everything was super. I wonder why he wakes me up from a lucid dream. Then I lose lucidity." The dreamer did not report receiving or answering any math problems.

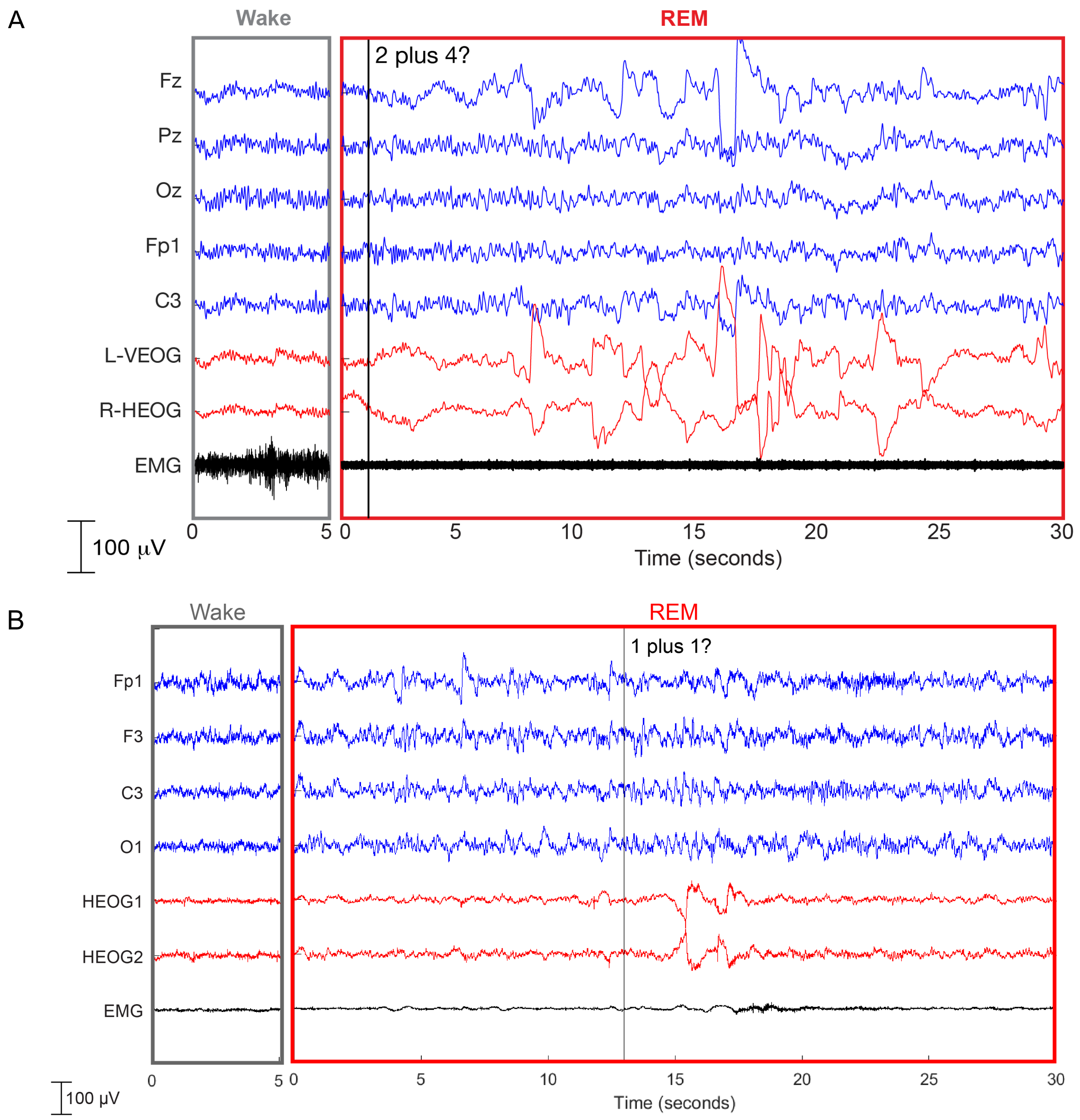

Figure S2. Ambiguous Responses, Related to STAR Methods.

A. An example of a math trial categorized as an “ambiguous response.” Raters agreed that a response had been given, but they did not agree on the response count. Upon awakening, the dreamer reported, “I remember one of the questions. That was when I think I’m having something cross my mind, and I don’t remember the details. I just remember I was solving a math problem with my eyes I think.”

B. Another example of a math trial categorized as an “ambiguous response.” Whereas two raters said there was a possible response, two raters said no response. Upon awakening, the participant reported, “I was still trying to fall asleep and did hear you, um, give a few calculations… but I still felt like I was, um, awake […] but I do have the feeling that I have been laying here again for quite some time… I don’t think I was dreaming, that is the feeling I had, so I don’t think I was aware of it. I did hear your voice a few times, yes, but then right before someone woke me up. I heard you whisper a little bit in the background, but that was just while I was laying here in bed—so to say—so not, um, not in a dream or something. I heard a calculation, but I don’t remember which numbers were in there.”

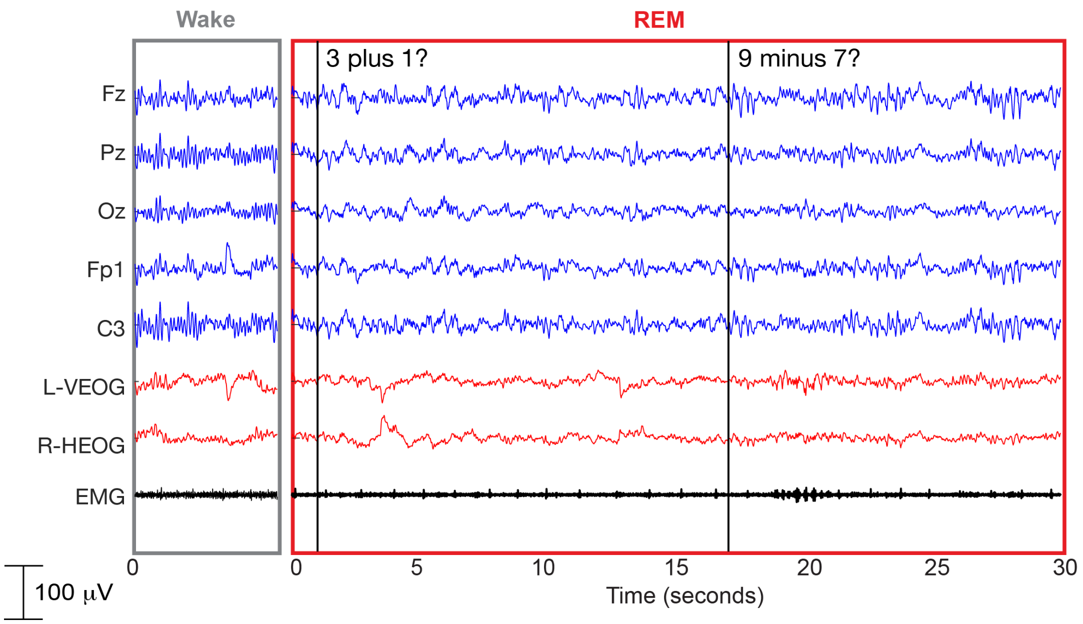

Figure S3. No Response, Related to STAR Methods. An example of two math trials categorized as "no response." Scorers agreed that no response was given. Upon awakening, the participant reported, "You were talking to me in my dream, and then you came up and said wake up." The experimenter asked if the participant remembered anything else, and the participant said, "I thought I heard you in the distance, but I couldn't hear them loud enough for the math questions. But I couldn't hear, I thought I said 1, but I actually said it [out loud]."

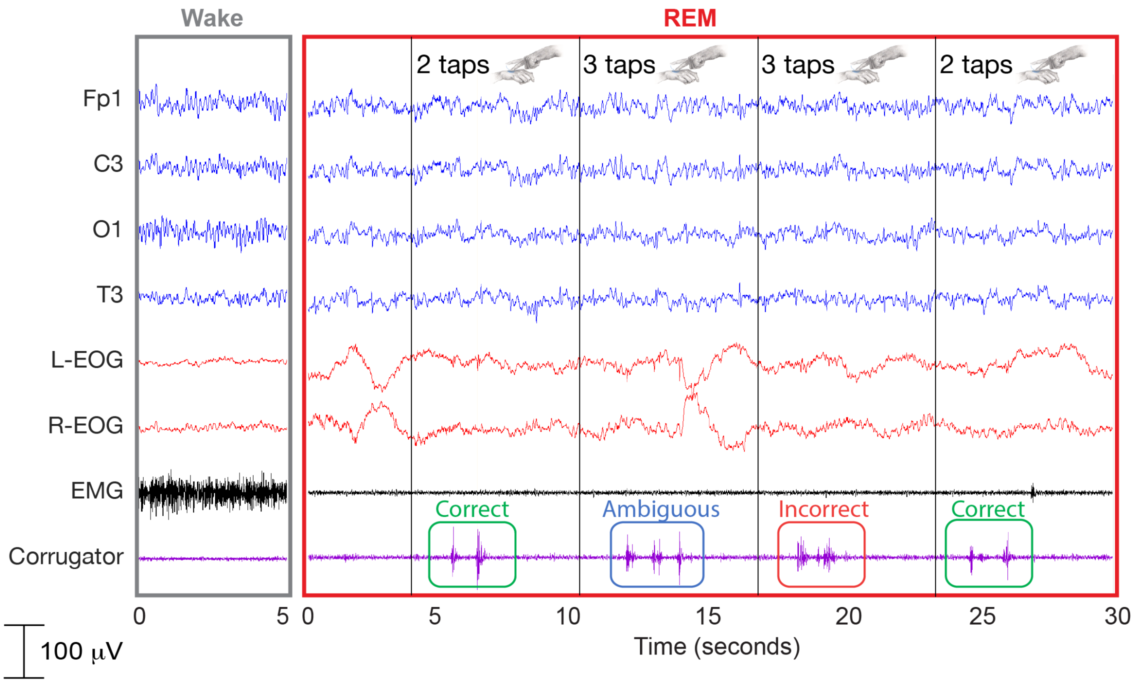

Figure S4. Responses in the Tactile Task, Related to STAR Methods. Examples of tactile-task responses considered correct, incorrect, and ambiguous. The participant was instructed to contract his corrugator the number of times he was tapped on the hand. Upon awakening, the participant reported, "I left the building and did some stuff I cannot remember right now. During the finger tapping, I was fighting against goblins with chopper hands. I remember being surprised I was able to do so many things at the same time as the task."

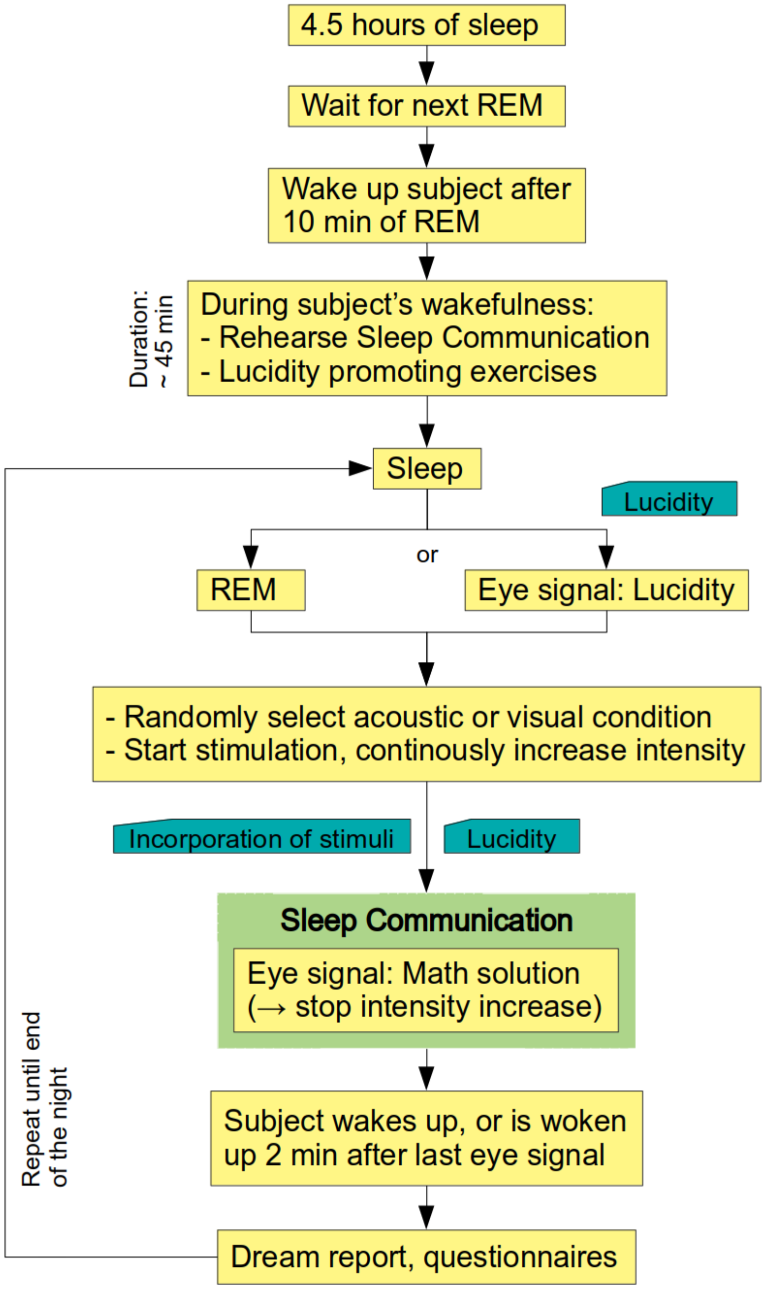

Figure S5: **Procedure for the German Team, Related to STAR Methods.**

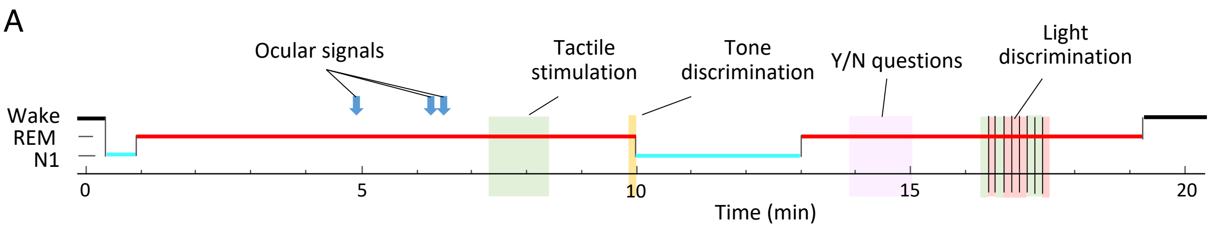

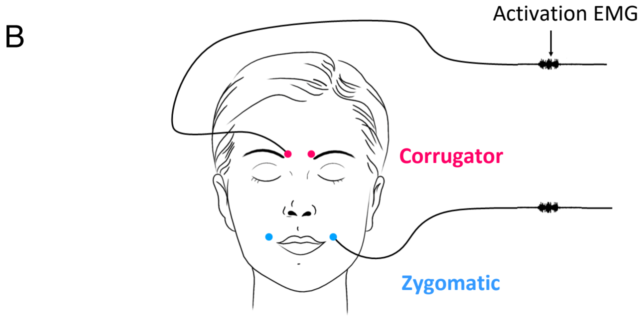

Figure S6. Procedure for the French team, Related to STAR Methods. (A) After the participant indicated lucidity with an LRLR eye signal, we attempted two-way communication in various ways. The first REM sleep period ended because of awakening due to the tones. **(B)** To communicate, participant AC contracted facial muscles. We recorded the signal via EMG.

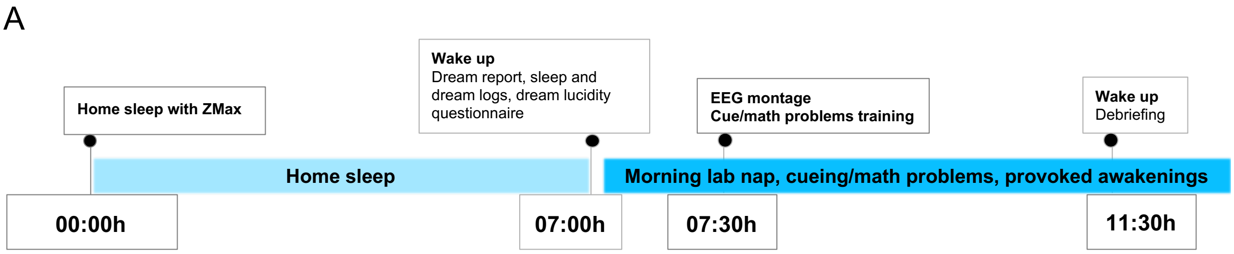

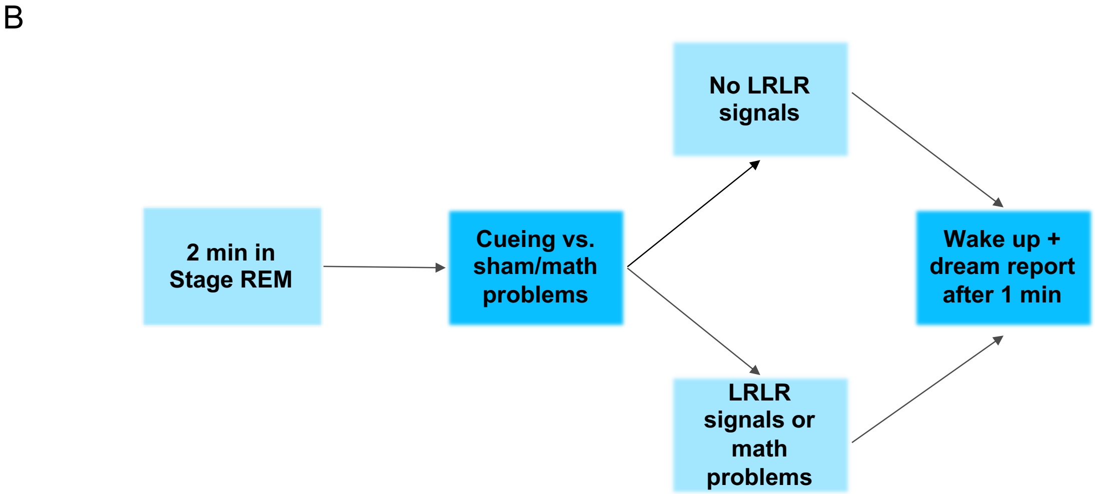

Figure S7. Procedure for the Dutch team, Related to STAR Methods. (A) Experimental procedure for overnight and morning nap protocol. **(B)** Procedure for cue and math problems administration.
